# Personalized Assessment of the Coronary Atherosclerotic Arteries by Intravascular Ultrasound Imaging: Hunting the Vulnerable Plaque

**DOI:** 10.3390/jpm9010008

**Published:** 2019-01-24

**Authors:** Theodore G. Papaioannou, Charalampos Kalantzis, Efstratios Katsianos, Despina Sanoudou, Manolis Vavuranakis, Dimitrios Tousoulis

**Affiliations:** 1 Biomedical Engineering Unit, First Department of Cardiology, Hippokration Hospital, Medical School, National and Kapodistrian University of Athens, 11527 Athens, Greece; bdkalantzis@gmail.com (C.K.); stratiskatsianos@yahoo.com (E.K.); drtousoulis@hotmail.com (D.T.); 2 Fourth Department of Internal Medicine, “Attikon” Hospital, Medical School, National and Kapodistrian University of Athens, 12462 Athens, Greece; dsanoudou@gmail.com; 3 Third Department of Cardiology, Medical School, National and Kapodistrian University of Athens, 11527 Athens, Greece; vavouran@otenet.gr

**Keywords:** coronary arteries, atherosclerosis, precision medicine, interventional cardiology

## Abstract

The term “vulnerable plaque” is commonly used to refer to an atherosclerotic plaque that is prone to rupture and the formation of thrombosis, which can lead to several cardiovascular and cerebrovascular events. Coronary artery atherosclerosis has a wide variety of different phenotypes among patients who may have a substantially variable risk for plaque rupture and cardiovascular events. Mounting evidence has proposed three distinctive histopathological mechanisms: plaque rupture, plaque erosion and calcified nodules. Studies have demonstrated the characteristics of plaques with high vulnerability such as the presence of a thin fibrous cap, a necrotic lipid-rich core, abundant infiltrating macrophages and neovascularization. However, traditional coronary angiographic imaging fails to determine plaque vulnerability features, and its ability to individualize treatment strategies is limited. In recent decades, catheter-based intravascular ultrasound imaging (IVUS) modalities have been developed to identify vulnerable plaques and ultimately vulnerable patients. The aim is to individualize prediction, prevention and treatment of acute coronary events based on the identification of specific features of high-risk atherosclerotic plaques, and to identify the most appropriate interventional procedures for their treatment. In this context, the aim of this review is to discuss how personalized assessment of coronary atherosclerotic arteries can be achieved by intravascular ultrasound imaging focusing on vulnerable plaque detection.

## 1. Introduction

Cardiovascular diseases are the number one cause of death worldwide, and the most frequent is coronary artery disease (CAD). Acute coronary syndrome (ACS), a common complication of coronary artery disease, is a term used to describe a range of conditions where the blood supply to any part of the heart is suddenly reduced or blocked. Atherogenesis, the triggering factor of ACS, is a chronic and developing inflammatory condition in which atheromas (or “plaques”) are developed in the inner lining (the intima) of arteries. Over time, the plaques harden, narrow the lumen of the arteries and restrict the blood flow. Acute coronary syndrome is an umbrella term encompassing the following clinical disorders: ST-segment elevation myocardial infarction (STEMI), non-ST-segment elevation myocardial infarction (NSTEMI) and unstable angina [1,2,3,4,5,6,7].

The term “vulnerable” is defined as “susceptible to injury or susceptible to attack”, suggesting that an event has high conditional probability to occur in the future. The source of the majority of ACS cases is the erosion or rupture of an atherosclerotic plaque, which leads to the development of intraluminal thrombus that can further limit, or even block, the blood flow. Thus, the term “vulnerable plaque” is often used to denote a plaque prone to rupture [8,9,10,11,12].

Nowadays, we are able to visualize detailed anatomical features of coronary arteries due to the evolution of high-resolution, intracoronary ultrasound imaging modalities, helping us to broaden our insight regarding the mechanisms responsible for atherogenesis and intravascular thrombus formation. Although several other intravascular modalities are now available for coronary artery assessment, such as optical coherence tomography (OCT), catheters for fractional flow reserve (FFR) measurement, near-infrared spectroscopy and so on, the present review aims to provide a brief overview assessing coronary atherosclerosis and plaque vulnerability by intravascular ultrasound (IVUS) imaging. Furthermore, we aim to highlight the contribution of IVUS in making the diagnosis of coronary atherosclerosis severity, and risk of coronary events, more precise by detecting plaque features that could not otherwise be assessed by traditional, routine angiographic examination (still the gold-standard method for coronary artery examination). Additionally, the significant advances in therapeutic precision introduced by the IVUS guidance of percutaneous coronary interventions (PCI) compared to angiographic-guided PCI are discussed.

## 2. Vulnerable Plaque

As mentioned above, acute coronary syndromes occur when atherosclerotic plaques rupture and occlusive thrombus is formed [4]. Autopsy findings have revealed that the main substrates for coronary thrombosis are in most cases plaque rupture and plaque erosion, while another potential mechanism including calcified nodules has also been demonstrated [10,11,12]. In general, a vulnerable plaque is characterized as an atherosclerotic plaque with microcalcifications, neovascularization, a thin fibrous cap and a large necrotic lipid-rich core with plentiful inflammatory cells and few smooth muscle cells. The histological hallmark of vulnerable plaque is the thin-cap fibroatheroma (TCFA); therefore, early recognition of TCFA might contribute to the development of interventional and pharmacological strategies to prevent plaque rupture [13,14,15,16,17].

Autopsy studies have shown an inverse relationship between the thickness of the cap and the risk of plaque rupture, with 95% of plaque rupture occurring in cases with a cap thickness of <65 μm. In a study by Narula et al., the significance of various pathological features in the distinction of TCFA from the fixed atherosclerotic plaque was analyzed and it was shown that the most reliable discrimination criterion is the thickness of the fibrous cap [18]. The existence of central necrosis in a large plaque adversely affects its stability. Expansion of the necrotic core can lead to thinning of the fibrous cap and therefore to plaque erosion. Indeed, autopsy studies have shown that ruptured plaques are associated with a large necrotic nucleus relative to non-ruptured plaques. Broken fibrous caps appear more often infiltrated by foamy macrophages than non-ruptured vulnerable plaques. Also, macrophages in the fibrous cap play an important role in thinning and ultimately rupturing the cap, secreting proteolytic enzymes such as matrix metalloproteinases [19].

Coronary arteries are likely to respond to the atherosclerotic process by dilatation or restriction of the arterial wall, defined as positive and negative remodeling, respectively. While vulnerable coronary artery plaques are associated with positive remodeling, stable plaques are most commonly related to negative remodeling of the vessel wall. Although by positive remodeling the vessel lumen may widen, continuous growth of the plaque into the interior of the lumen can induce neovascularization from small vessels originating from a network of vessels that irrigate the outside layer. It has been found that the development of these neovessels negatively affects plaque stability. They cause hemorrhages inside the plaque, which are due to the fact that these neoplastic vessels are fragile, with a lack of adequate supportive cells [20].

Calcium deposition in atherosclerotic plaques is associated with all stages of atherosclerosis and may occur even in the initial phase of intima thickening. The presence of microcalcifications within the thin fibrous cap or a particular calcification pattern called “spotty calcification” can be an indicator of plaque vulnerability. In contrast, larger calcium deposits, which consist of the joining of smaller regions of microcalcification, are associated with greater plaque stability. Therefore, although the presence of microcalcification in the fibrous cap and spotty calcification have been related to plaque rupture, plaques composed primarily of large calcification deposits are considered to be more stable [13,14,15,16].

### 2.1. Intravascular Ultrasound: IVUS

Intravascular ultrasound is an invasive imaging technique using a catheter that allows real-time, longitudinal, cross-sectional evaluation of the vessel and lumen dimensions, as well as the distribution and morphology of atherosclerotic plaques. IVUS grayscale images are constructed by measuring the intensity and timing of reflected signals, with an axial analysis of 150–250 μm with a 45 mm penetration depth.

Intravascular ultrasound uses a small-sized ultrasound probe placed at the end of a flexible (2.5–3.5 French) catheter, which is inserted into the coronary arteries. The standard operating frequencies of IVUS catheters are 20–60 MHz [21,22]. The safety of IVUS has been demonstrated in several studies. With the exception of transient vascular spasm, the incidence of complications due to IVUS is extremely low (less than 0.6%), while no acceleration of the atherosclerosis process has been observed with its use.

**Figure 1:**
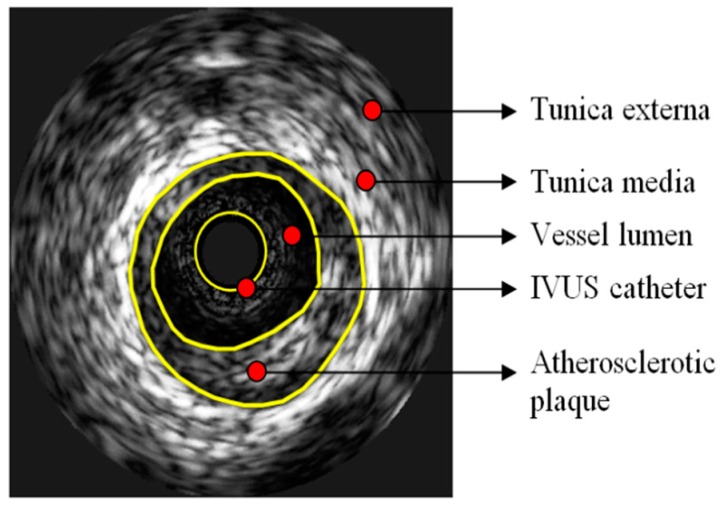
Typical conventional grayscale display of an image acquired through intravascular ultrasound imaging (IVUS). Hippokration Hospital, 1st Department of Cardiology, National and Kapodistrian University of Athens.

In the classical intracoronary ultrasound based on grayscale, healthy coronary arteries display three distinct layers. Atherosclerotic plaque is formed in the first layer, the intima. The second layer is the middle tunica, which is opaque and consists of smooth muscle cells. The third layer distinguished in the IVUS consists of the acoustic surface between the outer elastic waist and the outside area (Figure 1). IVUS grayscale cannot be used to identify and quantify specific histological features, as ultrasound images are radically different from histological. Atherosclerotic plaques with a rich adipose nucleus appear to have less echogenicity than the surrounding adventitia, while fibrous cap atheromatic plaques posses the majority of atherosclerotic lesions and show moderate echogenicity. On the other hand, calcified plaques have increased echogenicity and produce sounds with acoustic shadowing [23].

### 2.2. Ruptured Plaque

Using IVUS, a ruptured plaque is defined as the cavity formation in contact with the lumen of the vessel with a broken-surface fibrous cap [24]. Rupture of an atherosclerotic plaque is the most common cause (60–70%) of ACS and is associated with poor prognosis [25]. Kusama et al. reported that plaque rupture is related to a higher incidence of heart attacks and higher non-reperfusion rates after percutaneous coronary intervention (PCI) [26]. A recent study using intravascular optical coherence tomography (OCT) and IVUS by Soeda et al. showed that non-reperfusion is associated with a negative outcome, including mortality and systolic dysfunction [27]. However, rupture of the atherosclerotic plaque, as identified by IVUS, does not represent the culprit atherosclerotic lesion. Hong et al. reported that IVUS detects atherosclerotic ruptured plaque in 66% of suspected lesions and in 17% of arteries not typically associated with acute myocardial infarction in patients following a heart attack [28,29]. Rupture of the atherosclerotic plaque associated with small minimum lumen area (MLA) and thrombus formation usually causes symptoms, whereas plaque rupture without symptoms is associated with the formation of scar tissue and the progression of atherosclerotic lesion.

### 2.3. Calcification

The extent of coronary artery calcification correlates with the degree of atherosclerosis and future cardiovascular events. Calcium is a potent reflector of ultrasound and, consequently, ultrasound beams do not enter or even penetrate calcium. As a result, calcium casts a shadow over deeper arterial structures [30]. The IVUS signature of calcium (or occasionally of dense fibrous tissue) is echodense (hyperechoic) plaque that is brighter than the reference adventitia with shadowing [30]. Unstable lesions are accompanied by the presence of calcified nodules [30]. Moreover, a small extent of calcium deposition with an arc of <90° is associated with advanced atherosclerosis and an acceleration in the progression of the disease [31]. IVUS is the most accurate method for detecting calcium; nonetheless, it may not detect calcium segments that are (hidden) deeper, behind large necrotic cores [32]. The presence of calcium has been shown to be associated with acute complications following invasive techniques such as bleeding, stent thrombosis (ST), vascular target revascularization (TVR) and myocardial infarctions following reconstitution. This can be explained by abnormal stent deployment and increased adverse effects following PCI (non-reperfusion, vascular separation, vascular perforation due to the use of high pressure for balloon deployment and atherosclerotic plaque modifiers), observed in atherosclerosis with a high degree of calcification. Therefore, IVUS can help to evaluate atherosclerotic plaque with greater precision compared to angiography, and can better guide the selection and the deployment of stents in order to achieve the best result in treating calcified lesions [33,34]. IVUS-guided PCI is associated with fewer complications and better patient outcomes, including a decrease in mortality compared to the classic procedure of PCI guided by angiography (as further discussed below). This reveals the important contribution of IVUS to the personalized/individualized assessment of atherosclerotic plaques by offering not only a higher quality of vessel and plaque images than angiography, but also a greater amount of quantitative measurements (i.e., structural, functional biomarkers and indices).

### 2.4. Virtual Histology–IVUS

**Figure 2:**
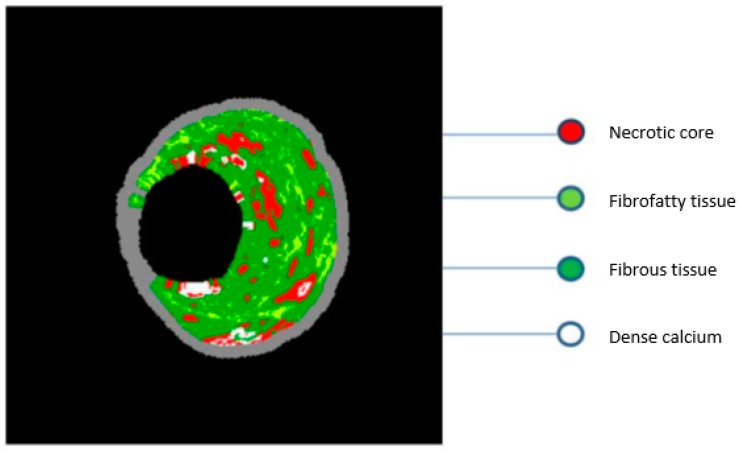
Intravascular ultrasound images analyzed by virtual histology algorithms (VH–IVUS). The components of the atherosclerotic plaque are fibrous connective tissue (dark green), fibro-fatty tissue (light green), necrotic core (red) and dense calcium (white).

Virtual histology–intravascular ultrasound imaging (VH–IVUS) uses advanced technology to analyze the frequency spectrum of reflected sound waves, and thus it can provide a more detailed analysis of the plaque composition (Figure 2). However, due to limited axial resolution (250 μm), VH–IVUS cannot directly determine the thickness of the fibrous cap, in contrast to OCT imaging (which is the gold-standard imaging modality for cap thickness measurement and TCFA assessment). Therefore, the definition of TCFA according to VH–IVUS is based on a lesion in which ≥10% of its composition refers to a necrotic core, without apparent fibrous tissue coverage and with an atherosclerotic burden of ≥40% [35]. The use of VH–IVUS to characterize components of the vulnerable plaque has been extensively investigated, and it has been shown that ACS-treated patients have significantly more TCFA plaques and more plaque fractures. In addition, culprit lesions in ACS patients had a higher percentage of necrotic core and less percentage of fibro-fatty components. In the PROSPECT (Providing Regional Observations to Study Predictors of Events in the Coronary Tree) study, 697 patients with ACS underwent VH–IVUS. During invasive angiography, both culprit lesions as well as lesions that are not considered suspicious were evaluated. The results showed that in the follow-up period, with a mean duration of 3 to 4 years, the re-manifestation of cardiovascular events was the same between the non-culprit (11.6%) and culprit atherosclerotic lesions (12.9%) [36]. Non-culprit plaques associated with the onset of cardiovascular events are characterized by a atherosclerotic load of ≥70% and a minimum lumen area of ≤4.0 mm^2^, or classified as TCFA based on VH–IVUS. Although VH–IVUS has been used in clinical research to identify vulnerable plaque characteristics, its ability to identify TCFA plaques and determine their quantitative characteristics remains challenging. An ex vivo study in pigs attempted to investigate the correlation between VH–IVUS and histological measurements of necrotic core size. The findings did not indicate a good correlation between VH–IVUS necrotic core measurements and histological findings [37]. This ex vivo study draws attention to the exclusive use of VH–IVUS measurements for assessing therapeutic approaches, predicting future events and testing the effectiveness of other imaging techniques.

## 3. Clinical Applications of IVUS

### 3.1. Assessment of Intermediate Coronary Lesions

Coronary angiography is the conventional method to assess the coronary anatomy and to search for stenotic lesions in the epicardial coronary vessels. Nonetheless, coronary angiography provides only a projection of the vessel lumen and cannot visualize the vascular wall or the detailed lumen geometry. The two-dimensional imaging of the coronary lumen, in addition to some technical features (vessel overlap, lesion foreshortening), can lead to inaccurate assessment of lesion severity. The evaluation of the hemodynamic significance of moderate coronary stenosis with 40% to 70% lumen reduction only using coronary angiography appears a difficult decision even for experienced interventional cardiologists [38]. Considering all this, it is well-known that the diagnostic precision of angiography is limited under specific circumstances. As IVUS can visualize the vessel wall and provide more-accurate vessel and lumen dimensions, it is therefore a useful, diagnostic tool that allows a more individualized and precise assessment of the extent and severity of the atherosclerotic disease, especially in the domain of angiographically intermediate stenosis.

### 3.2. Non-Left Main Lesions

Fractional flow reserve (FFR) is an optional method for the hemodynamic evaluation of intermediate lesions [39]. Nonetheless, several studies have described a good correlation between hemodynamic data acquired by FFR and anatomic information derived from IVUS. Specifically, a minimal lumen area (MLA) of 4 mm^2^ has been proposed from several studies as a safe cut-off value to identify significant coronary lesions. An MLA ≥ 4 mm^2^, with a diagnostic accuracy of 89%, showed a clear correlation with a normal coronary flow reserve >2.0 [40]. Another study of 53 intermediate lesions demonstrated a correlation of an MLA ≤ 4 mm^2^ with an abnormal FFR < 0.75, with a specificity of 56% and a sensitivity of 92%, respectively [41].

Nonetheless, assessment of the severity of a lesion dependent only on IVUS data could be erroneous and thus should be avoided. Numerous other factors, like lesion length, reference vessel diameters, area of the myocardium at risk and plaque burden should be co-evaluated when interpreting the acquired IVUS data. All this information (biomarkers) derived by IVUS may synthesize a more detailed and individualized “picture” of the atherosclerotic vessel leading to a more precise assessment of its vulnerability. Not surprisingly, studies on intermediate lesions in smaller vessels (reference diameter < 3.0 mm) showed a smaller MLA (≤2.0 mm^2^) to be correlated with an FFR < 0.75 [42]. In a similar population of patients with intermediate lesions in smaller vessels, an extended approach combining MLA with other anatomic factors (lesion length and plaque burden) showed the strongest correlation with FFR < 0.75 [43].

In clinical practice, an MLA ≥ 4 mm^2^ can safely identify non-ischemic lesions without the need for revascularization, while in the case of an MLA ≤ 4 mm^2^ other modalities such as FFR may be used and anatomic (lesion length) and pathophysiologic factors (plaque burden) should be also taken into consideration [44].

### 3.3. Left Main Disease

Anatomic factors (short segments, varying angles of the left main bifurcation, overlapping branches, etc.) as well as technical factors (the aorto-ostial junction is usually non-perpendicular to the lumen axis) make the interpretation of left main disease angiographically difficult, thus necessitating the use of additional, more-precise diagnostic tools. Not surprisingly, angiographic assessment of the left main coronary artery (LMCA) shows the greatest interobserver variability [45]. A multicenter, prospective study of 354 patients with intermediate lesions in unprotected LMCAs used an MLA of 6 mm^2^ as a cut-off for revascularization. After 2 years, only 8 patients (4.4%) from the deferred group (MLA > 6.0 mm^2^) underwent revascularization, none of them in the setting of acute coronary syndrome [46]. It should be noted that this cut-off value seems to be population-dependent. An MLA > 6 mm^2^ should be considered as a criterion of a non-significant LMCA without need for intervention. Nevertheless, an MLA < 6 mm^2^ should not be exclusively regarded as an indication for revascularization without taking into account other clinical and angiographical parameters. In such a scenario, FFR or a noninvasive stress test should be performed [44].

### 3.4. IVUS-Guided Interventions

Recently, the European Association of Percutaneous Cardiovascular Interventions published an updated expert consensus document on the clinical use of intracoronary imaging for guidance and optimization of coronary interventions [47]. In the context of better understanding the diverse mechanisms of suboptimal stent deployment, such as stent under-expansion, incomplete stent apposition, smaller minimal stent area, incomplete lesion coverage and persistent edge dissections [48,49,50,51], IVUS-guided percutaneous coronary intervention (PCI) has been associated with less adverse cardiac effects. This evidence highlights the significant improvement in therapeutic (besides diagnostic) precision of coronary atherosclerotic disease achieved by IVUS.

In the domain of bare metal stents (BMSs), several studies have demonstrated the clinical value of IVUS guidance for individualizing and optimizing the interventional result through reduction of restenosis and target vessel revascularization (TVR). In a meta-analysis of 2193 patients from seven randomized trials, an IVUS-guided interventional strategy in patients treated with BMSs reduced TVR by 13% compared with the group of angiography-guided PCI [52].

Similar results have been noted in the era of drug-eluting stents (DESs) for IVUS-guided interventions of LMCAs as well as for non-left main disease. A large study of 1670 patients with LMCA lesions, treated with DESs, was undertaken to investigate the value of IVUS guidance in interventions. After a mean follow-up period of three years, the group of patients who underwent IVUS imaging showed significantly reduced incidence of major adverse cardiac events (MACE) and TVR compared to those who received angiographic guidance [53].

Furthermore, the chronic total occlusions (CTO)–IVUS trial [54] and the IVUS–XPL trial (lesion length ≥ 28 mm) [55] demonstrated a significant reduction in MACE under IVUS guidance. Both trials benefited from a reduced rate of restenosis and a resulted decrease in repeat revascularization.

Similarly, a meta-analysis of seven randomized controlled trials following 3192 patients treated with DESs confirmed the benefits of IVUS-guided intervention in regards to MACE, cardiovascular mortality and stent thrombosis after a follow-up of 12 to 24 months [56]. However, this meta-analysis had some limitations. First, most individual trials showed a favorable statistical trend rather than a significant superiority of IVUS guidance, a fact that limits the power of the analysis. The heterogenicity of the treated lesions, including non-complex lesions, and the lack of prespecified guidance protocols represent further limitations.

Regarding the impact of the lesion complexity and the clinical setting under which IVUS was performed, a large observational study including 8583 patients demonstrated the most pronounced benefit of IVUS use in patients with ACS and complex lesions (ADAPT DES) [57]. A similar benefit of IVUS guidance in ACS patients and complex lesions regarding MACE and mortality was shown in a meta-analysis of 20 studies [58].

The above findings were confirmed in the most recent meta-analysis of a total of 31 studies including of 17,882 patients. IVUS-guided PCI was associated with a significantly reduced risk of all-cause death, myocardial infarction, target lesion revascularization and stent thrombosis [59]. Notably, IVUS-guided intervention has been associated with lower doses of iodine contrast agent and therefore lower incidence of contrast-induced kidney injury [60].

## 4. IVUS in Various Stages of Coronary Intervention

### 4.1. Prior to Intervention, Selection of Optimal Stent Sizing

Prior to intervention, IVUS can assess plaque composition and distribution and, according to this large amount of quantitative and qualitative information, the treatment strategy can be modified and individualized according to the specific patient’s characteristics and therefore the appropriate stent size and length can be selected. In cases of plaques with extensive calcification, a more aggressive therapy, like rotational atherectomy, may be preferred, whereas a lipid-rich plaque may require a less aggressive approach to avoid distal lipid embolization. Optimal stent sizing (stent diameter and length) plays a crucial role in early and long-term interventional results. Among various factors, stent under-expansion is a strong predictor of early stent thrombosis and restenosis [61,62]. Several approaches have been described for IVUS-based appropriate-stent-diameter selection. In common clinical practice, the use of distal lumen diameter has emerged to a safe, reproducible option. Specifically, the mean distal lumen diameter with up rounding the stent (0–0.25 mm) can be safely used. Further, appropriate definition of the landing zone and especially avoidance of landing within an area of residual plaque burden has been associated with reduced incidence of restenosis [63]. Hence, IVUS-facilitated optimal stent sizing can lead to a larger stent diameter and greater minimal stent area [64].

### 4.2. Optimization after Stent Implantation

After stent implantation, IVUS can identify correctable abnormalities, such as under-expansion, malposition and edge dissection. A minimum stent cross-sectional area of 5.5 mm^2^ for non-left main lesions, 7 mm^2^ for distal left main disease and 8 mm^2^ for proximal left main disease should be targeted [65,66]. Malposition is defined as lack of contact between stent struts and the vessel wall. It can occur acutely, but it can also appear later as a result of an underlying vascular process. The clinical relevance of acute malposition remains debatable [67]. However, extensive malposition should be avoided and corrected, if technically feasible. In contrast to acute malposition, late malposition can lead to stent thrombosis [68]. Large-edge dissections as well as intra- and extramural hematomas are also detectable abnormalities [69].

### 4.3. Assessment of the Mechanism of Stent Failure

IVUS can be applied to investigate the mechanism of stent failure. The European Society for Cardiology (ESC) guidelines on myocardial revascularization recommended the performance of intracoronary imaging by IVUS in case of restenosis or stent thrombosis with a Class IIa recommendation [70]. The most common cause of restenosis is neointimal hyperplasia, followed by chronic stent under-expansion, stent fracture and neoatherosclerosis, commonly >1 year after DES implantation [71,72]. In early stent thrombosis, uncovered struts and malposition were the leading abnormalities, whereas late stent thrombosis was predominantly caused by malposition and neoatherosclerosis [68,69,70,71,72,73].

### 4.4. New Developments in IVUS Imaging

**Figure 3:**
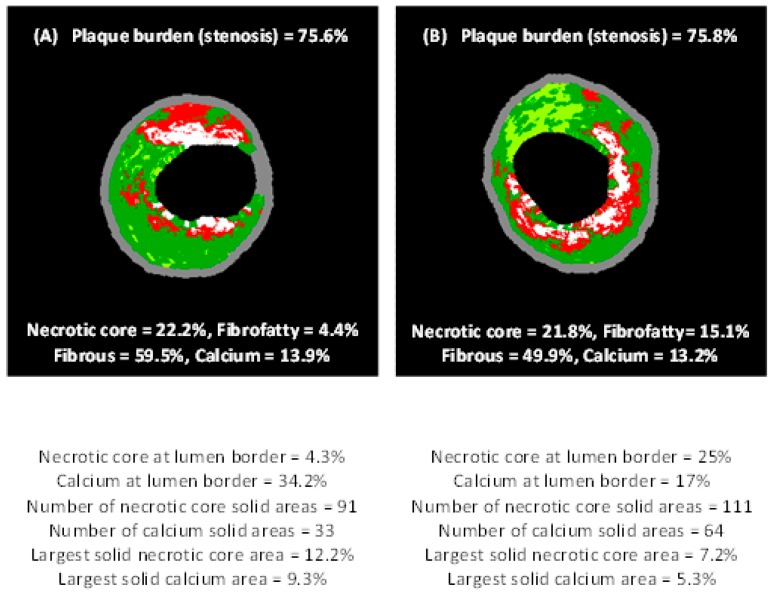
Two atherosclerotic plaques with similar percentages of stenosis, necrotic cores and calcium areas. Images were acquired through virtual histology–intravascular ultrasound imaging (VH–IVUS). (**A**) Plaque with less necrotic core and more calcium adjoined to the vessel lumen. A lower dispersity of necrotic core (NC) and calcium (C) is apparent. (**B**) In contrast to case (**A**), more necrotic core and less calcium are adjoined to the vessel lumen, whereas a greater dispersity of NC and C is apparent. Images were published by Papaioannou et al. [77].

Besides the classic applications of IVUS mainly targeting geometrical and structural features of atherosclerotic lesions, specific computational methods and algorithms have been developed that provide extra information concerning plaque vulnerability. Neovascularization (or neoangiogenesis) is a well-know process which might be more or less apparent in atherosclerotic lesions and is related with plaque progression and vulnerability. Combining contrast-enhanced IVUS imaging (using microbubbles as a contrast agent) and specific computational algorithms, it has been found that visualization and semi-quantification of the presence and density of vasa vasorum and neovessels within and around a coronary atherosclerotic lesion are feasible [74,75,76]. Moreover, a step forward in VH–IVUS (which has already been used for more than a decade) has been introduced as a new computational post-hoc analysis of VH–IVUS imaging that determines more than 30 new compositional and structural biomarkers. These biomarkers indicate spatial distribution, heterogeneity and dispersity of each VH–IVUS-derived component within the plaque area and also with respect to the plaque-lumen border [77,78], as schematically illustrated in Figure 3. However, this new imaging technique [77] has not been validated by histopathology yet.

## 5. Conclusions

The rapid and emerging developments in biomedical engineering and computer science have a tremendous impact in medical imaging and particularly in intravascular modalities. More information and knowledge is continuously gained concerning not only atherosclerotic plaque phenotypes (i.e., geometry), but also about in vivo atherosclerotic processes and plaque progression. The plethora of information and the new computed structural, compositional and functional biomarkers using IVUS and IVUS-based modalities is valuable for the personalization of diagnosis and guidance of interventions (i.e., stenting) for the optimal management of patients with coronary artery disease. Nonetheless, more analyses of cost effectiveness remain to be conducted to further explore the use of IVUS modalities in clinical practice and determine those cases where IVUS will be most beneficial for patients’ outcomes.
